# Epidemiology of neurocognitive disorders in adults from urban-marginalized areas: a door-to-door population-based study in Puente Piedra, Lima, Peru

**DOI:** 10.3389/fpubh.2023.1228008

**Published:** 2023-10-19

**Authors:** Eder Herrera-Perez, Nilton Custodio, Monica Diaz, Rosa Montesinos, Alexandra Chang, Mirla Villafuerte, Serggio Lanata

**Affiliations:** ^1^Vicerrectorado de Investigación, Universidad San Ignacio de Loyola, Lima, Peru; ^2^Unidad de Investigación, Instituto Peruano de Neurociencias, Lima, Peru; ^3^Centro de Excelencia en Biotecnología e Investigación Traslacional, Lima, Peru; ^4^Escuela Profesional de Medicina Humana, Universidad Privada San Juan Bautista, Lima, Peru; ^5^Department of Neurology, University of North Carolina at Chapel Hill, Chapel Hill, NC, United States; ^6^Facultad de Ciencias de la Salud, Universidad Peruana de Ciencias Aplicadas, Lima, Peru; ^7^Edgardo Rebagliati Martins National Hospital (EsSALUD), Lima, Peru; ^8^Department of Neurology, Memory and Aging Center, University of California, San Francisco, San Francisco, CA, United States

**Keywords:** neuroepidemiology, mild neurocognitive disorder, major neurocognitive disorder, cognitive impairment, dementia, mild cognitive impairment, door-to-door study

## Abstract

**Background:**

In Latin America (LA), the prevalence of dementia is expected to triple to 150 million people by 2050. The 2020 Lancet Commission report identified several modifiable dementia risk factors, yet few social and environmental factors, most relevant to vulnerable regions of LA, were highlighted in this report. We sought to assess the epidemiology of neurocognitive disorders (NCD) in Puente Piedra, one of the most socially and economically vulnerable districts of Lima, the capital of Peru.

**Methodology:**

This was a cross-sectional door-to-door observational study that used two-stage household sampling. One young adult (30–59 years) and one older adult (>60 years) per household were enrolled. We collected demographic, clinical, and neurocognitive data. Addenbrooke’s Cognitive Examination (young adults) and the RUDAS-PE (older adults) were used, classifying participants as cognitively normal, possible mild NCD, or possible major NCD.

**Results:**

We enrolled 247 participants (median age 46 years; 67% female). One-fourth had not completed secondary school and more than 50% completed only secondary school. Most participants were housewives (46%) and 21% did not have health insurance. The overall prevalence of possible NCD was 30% (25.6 and 41.8% among younger adults and older adults, respectively). Among younger adults, those ages 55–59 years more frequently had NCD (70%) compared to younger age ranges. Among older adults, only 3 subjects (4.5%) had major NCD.

**Conclusion:**

We found a high frequency of possible NCDs in a socially and economically vulnerable community in Lima, Peru, with younger adults showing levels of NCD higher than expected. Our findings support the need for health systems to incorporate cognitive screenings programs for NCD in younger ages. Future research on NCD would include younger populations, particularly in vulnerable communities.

## Introduction

Preserved cognitive health is arguably the most important determinant of an individual’s quality of life (1). Complex attention skills, executive functions, expressive and receptive language abilities, perceptual-motor functions, learning and memory, and social cognition skills (2), are all essential to an individual’s activities of daily living (ADLs) and attainment of their long-term goals.

Neurodegenerative and vascular brain disorders are the most common causes of cognitive impairment (CI) in adults, with Alzheimer’s disease (AD) being the most common neurodegenerative disorder worldwide. Persons with CI due to neurodegenerative and vascular disease commonly experience long durations of illness, often lasting decades, beginning with subjective cognitive decline, progressing to mild cognitive impairment (MCI), and culminating with dementia in late stages of illness (3, 4). Whereas MCI is broadly characterized by CI that does not significantly interfere with ADLs, dementia is characterized by decline in at least one cognitive domain (5) that leads to significant loss of independence of ADLs (6). The DSM-5 describes MCI and dementia as mild and major neurocognitive disorder (NCD), respectively (5, 7–9), and outlines clinical diagnostic criteria for each.

According to the WHO, about 50 million people were living with dementia worldwide in 2015, with 60–70% due to AD. It is estimated that 5–8% of the general population over 60 years of age currently lives with dementia, with 63% being from low-to-middle-income countries (LMIC) (10). In Latin America (LA), a systematic review of 31 publications estimated a dementia prevalence of 11% in older adults, with higher prevalence among women and persons living in urban areas (11).

Researchers have identified several potentially modifiable risk factors that account for an estimated 40% of cases of dementia worldwide (12) and up to 56% of cases of dementia in LA (13). Most of these risk factors represent downstream medical conditions (hypertension, diabetes, etc.) and behavioral exposures (physical inactivity, social isolation, etc.). Research on the cognitive impact of more upstream socioeconomic vulnerability factors, which are highly prevalent in LMIC, is relatively lacking. In this study, we sought to conduct a cross-sectional door-to-door study assessing the prevalence of NCD among both younger and older adults in the district of Puente Piedra, located in Lima, the capital city of Peru, in order to determine the prevalence of NCD across all adult ages in this vulnerable community.

## Materials and methods

### Study design and setting

**Figure 1:**
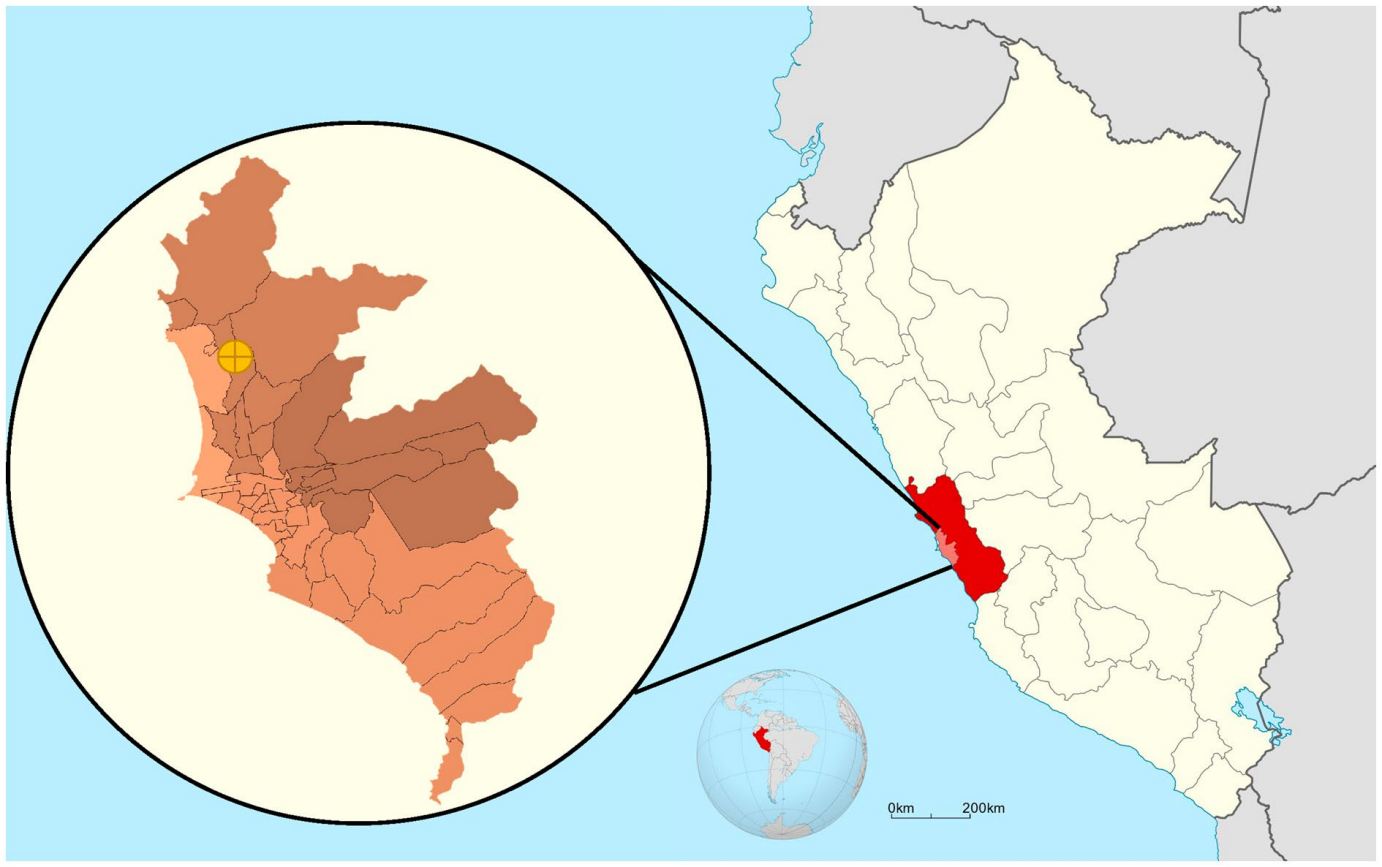
Map of the capital city of Lima, Peru, with zoomed area of the Puente Piedra district.

We conducted a cross-sectional door-to-door study in the district of Puente Piedra, located in Lima, the capital city of Peru, in March 2022 (Figure 1).

### Study population

We included households in which at least one Spanish-speaking adult ages 30 years or older was available at the time of the home visit, had been living in the home for at least 6 months, and was able to provide informed consent. We excluded households in which no person was eligible to participate in study procedures due to the following: serious physical (e.g., motor, visual, or hearing impairment) or mental (e.g., schizophrenia, cerebral palsy) condition that limited their ability to complete the neurocognitive testing. In each household, we interviewed up to two people: one young adult (age 30–59) and one older adult (age ≥ 60).

According to data from the “*Censos Nacionales 2017: XII de Población, VII de Vivienda y III de Comunidades Indígenas*” (14), in 2017 the district of Puente Piedra was home to 322,991 people: 76,380 were 30–44 years old, 54,920 were 45–64 years old, and 15,621 were ≥ 65 years old. The district spans 71.18km^2^, is divided into five zip codes, and has 86,421 dwellings distributed throughout its area.

### Study sample

In our sample design, clusters of homes were created on the block level. In order to obtain representative results of the population, we performed multi-stage probability sampling considering the blocks as clusters. In the first stage, we conducted simple random sampling (SRS) and considered each zip code of each district one cluster (or sampling unit). In the next and second stage, we identified all the dwellings within each cluster and selected the dwelling that would be visited using systematic random sampling. In case of absence or non-response in a dwelling, the dwelling was replaced by the one adjacent to it following a clockwise direction. Finally, in each household we conducted a simple random sampling among the eligible household members of each age group (young adults and older adults) to select one participant(s) within each group.

The present study covered two of the five postal codes into which the district of Puente Piedra is divided. Considering a total district population of 148,014 people aged 30 years or more, we applied calculation parameters (*p* = 6.85 and confidence = 0.95) to estimate the precision of the sample size of 247 participants, obtaining a precision value of 3.15. The calculations were performed with the Epidat v4.2 program.

We approached 268 households, 26 of which were excluded because of incomplete cognitive evaluation or age < 30 years.

### Study variables

Using structured questionnaires, we collected participant sociodemographic data (age, biological sex, education, among others) and health status of each participant (comorbidities, body weight and height, blood pressure (15), among others). We also collected sociodemographic data of each household, including data on overcrowding, defined as a three or more persons per room in the home (16). Socioeconomic level (SEL) of participants was defined using an adaptation of the methodology of the *Asociación Peruana de Empresas de Investigación de Mercado (APEIM)* (17), with the following definitions: SEL A, owns a car for personal use; SEL B, owns a laptop/PC for personal use and/or microwave oven; SEL C: owns a refrigerator and/or washing machine for personal use; SEL D: owns bathroom or toilet connected to public sewage system within the person’s home; SEL E: is unable to cover monthly cost for basic food and necessities.

We performed depression screening using the Patient Health Questionnaire (PHQ-9) with the following cut-off scores: no depression (<4), mild depression (5–9), moderate depression (10–14), moderate–severe depression (15–19), and severe depression (≥20) (18, 19).

To assess the presence of possible NCDs, we administered two brief cognitive tests (BCTs): (1) *Addenbrooke’s Cognitive Examination* (ACE) (20–22) among the young adults; and (2) *Rowland Universal Dementia Assessment Scale* (RUDAS) in its Peruvian version (RUDAS-PE) (23–25) among the older adults. The ACE is composed of five subscales (attention, memory, language, verbal fluency, and visuospatial skills), with a maximum total score of 100 points (20). We used the version translated into Spanish and adapted by researchers from Chile and Argentina, which has previously been successfully applied in a Peruvian sample (21). The ACE was selected for use in younger adults because it has been previously validated and used in this population (26). The ACE distinguishes between cognitively healthy and NCD but does not distinguish between mild NCD and major NCD (20). The RUDAS-PE assesses five core neurocognitive domains (episodic memory, visuospatial functions, motor praxis, judgment, and language) with a maximum total score of 30 points. The RUDAS-PE has been validated by our team among Peruvian adults with illiteracy and low educational levels (27) and also among Peruvians with mid-level educational attainment (23). The RUDAS-PE distinguishes between cognitively healthy, mild NCD, and major NCD.

We evaluated the cognitive performance of each study participant using these BCTs, and determined each participant’s cognitive classification (healthy, possible mild NCD, possible major NCD) based on established cut-off scores (21, 23, 27) validated against gold standard DSM-V criteria (28).

### Study procedures

A training program was specifically designed for the field team that performed the in-home evaluations of participants. The training program was comprised of knowledge-based and practice sessions, both face-to-face and virtual, to appropriately and accurately train each evaluator to apply the tests selected for this study (ACE, RUDAS-PE) and the data collection methods in a standardized fashion. Each evaluator was trained in the following systematic fashion: (1) evaluators familiarized themselves with the instruments by completing unsupervised practice sessions; (2) evaluators underwent training in the proper administration of all instruments used (brief cognitive tests, PHQ-9, and data entry platform); (3) evaluators underwent structured didactic sessions with videos of real cases and practice sessions; and (4) real cases were evaluated by the trained evaluators and their scores were compared with the scores of experienced evaluators (gold standard). Thus, the evaluators who were selected as the research staff for this study were those evaluators whose scores were closest to the scores of the experienced evaluators.

We collected data on a mobile device platform that had been previously validated (29). Research staff conducted door-to-door visits in each of the pre-selected households. After providing written informed consent, the participant was interviewed and assessed by the trained field personnel. All household visits were conducted during the month of March 2022.

In order to maintain data accuracy, we completed periodic checks of the data by an independent reviewer who was not on the field team collecting data (EH). Thus, we identified and excluded the repeated observations (duplications) and incomplete registers.

### Statistical analyses

We performed analyses to estimate the community prevalence of possible NCDs overall and by age groups. For all descriptive statistics, we reported absolute and relative frequencies for categorical variables (e.g., neurocognitive status, sex, origin, etc.), and for continuous variables (e.g., age, education, performance on BCTs, etc.), we used median and interquartile range due to the non-normalized distribution of the data. All analyses were performed using STATA version 17.

### Ethical considerations

This study was approved by the ethics committee of the Universidad San Ignacio de Loyola. Written informed consent was requested according to standard procedures. Participants benefited from the provision of their individual test results, as well as relevant counseling if appropriate.

## Results

We visited a total of 222 clusters (blocks), where we enrolled at least one participant. Our study enlisted a total of 247 participants (median age 46 years [interquartile range (IQR) = 22]), 66.7% women and 64.23% were married or cohabitating. About 25% had not completed secondary school and at least half had completed schooling (11 years of education). Roughly 28% reported Lima as their region of origin and 17.4% reported a native language other than Spanish (Quechua and Aymara), although all participants were Spanish-speaking adults (inclusion criterion for this study). Nearly three out of four participants (74.8%) were classified as low socioeconomic level (SEL C, D, and E), but overcrowding was found in only 7.9% of households. The largest occupational group was housewives (46.3%) and 21.5% of respondents reported not having any health insurance coverage despite a national health insurance program in Peru (Table 1).

**Table 1:** Sociodemographic characteristics of a sample of adults from the district of Puente Piedra, Lima, Peru, 2022.

	*n* or mean	% or SD
Age in years* (*n* = 247)	46	22
Sex (*n* = 243)		
Male	81	33.33
Female	162	66.67
Marital status (*n* = 246)		
Married	71	28.86
Cohabitant	87	35.37
Single	56	22.76
Separated or divorced	13	5.28
Widowed	19	7.72
School education in years* (*n* = 236)	11	5
Highest level of education attained (*n* = 246)		
No education	6	2.44
Preschool	8	3.25
Primary	49	19.92
Secondary	136	55.28
Non-university higher education	36	14.63
Superior university	11	4.47
Origin (*n* = 245)		
Lima	71	28.69
Ancash	41	16.73
Cajamarca	27	10.76
Rest of the country	106	43.82
Native language (*n* = 247)		
Spanish	204	82.59
Quechua	43	17.41
Socioeconomic level - SEL (*n* = 247)		
SEL A	13	5.37
SEL B	48	19.83
SEL C	91	37.6
SEL D	39	16.12
SEL E	51	21.07
Employment status (*n* = 246)		
Housewife	114	46.34
Self-employed	38	15.45
Employee	57	23.17
Freelance	8	3.25
Retired	11	4.47
Another	3	1.22
No current employment	15	6.1
Person-room ratio (*n* = 242)	1.5	1
Overcrowding (*n* = 242)		
No	210	86.78
Yes	32	13.22
Health insurance (*n* = 247)		
No health insurance	53	21.46
“Seguro Integral de Salud” (SIS)	128	51.82
ESSALUD	56	22.67
Armed or police forces	3	1.21
Private health insurance	2	0.81
Do not know	5	2.02

(*): data shown as median and interquartile range. ESSALUD, Social Security Health Insurance program; SD, standard deviation; SIS, health insurance provided by the Ministry of Health.

**Figure 2:**
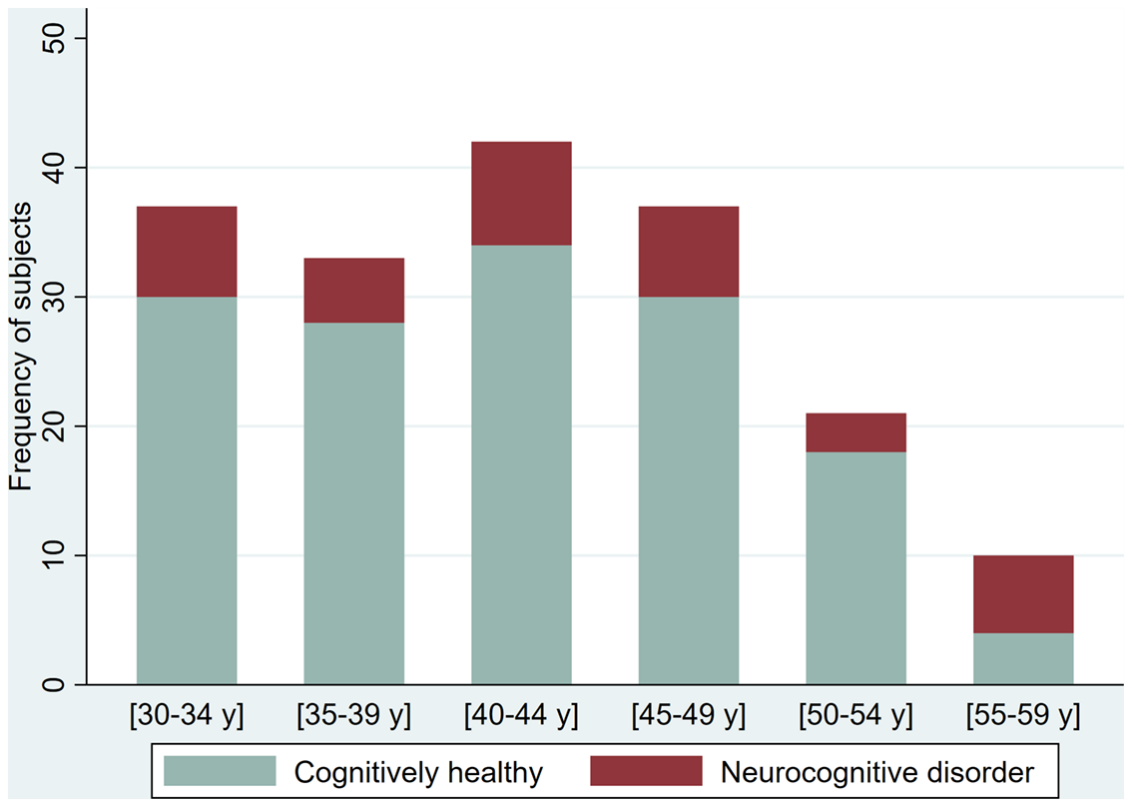
Frequency of cognitively healthy younger adults and younger adults with possible neurocognitive disorders, per age group.

The prevalence of possible NCD (mild NCD + major NCD) was 25.6 and 41.8% in younger adult and older adults, respectively (Table 2). Younger adults (30 to 59 years) in the oldest five-year age range had a higher frequency of NCD (70%, age 55–59 years) compared with younger age ranges in the group who had a NCD prevalence ranging from 21.2 to 24.3%. Among older adults (60 years and older), NCD prevalence increases by each five-year age groups (from 26.09 to 58.33%), with reversal of this pattern in the group 80–84 years of age (from 50 to 0%). In the group over 65 years of age, we identified only one case with major NCD (Figures 2 3).

**Table 2:** Neurocognitive profile of adults from the district of Puente Piedra, Lima, Peru, 2022.

	*n* or mean	% or SD
Younger adults (30–59 years old, *n* = 180)		
ACE* score	80	16
*Cognitive classification*		
Cognitively healthy	134	74.44
Neurocognitive disorder	46	25.56
Older adults (60 years and older, *n* = 67)		
Score obtained in RUDAS-PE*	24	4
*Cognitive classification*		
Cognitively healthy	39	58.21
Mild neurocognitive disorder	25	37.31
Major neurocognitive disorder	3	4.48
All adults (30 years and older, *N* = 247)		
*Cognitive classification*		
Cognitively healthy	173	70.04
Possible neurocognitive disorder	34	29.96

ACE, Addenbrooke’s Cognitive Examination; RUDAS-PE, Peruvian version of the Rowland Universal Dementia Assessment Scale (RUDAS-PE); SD, standard deviation.

**Figure 5:**
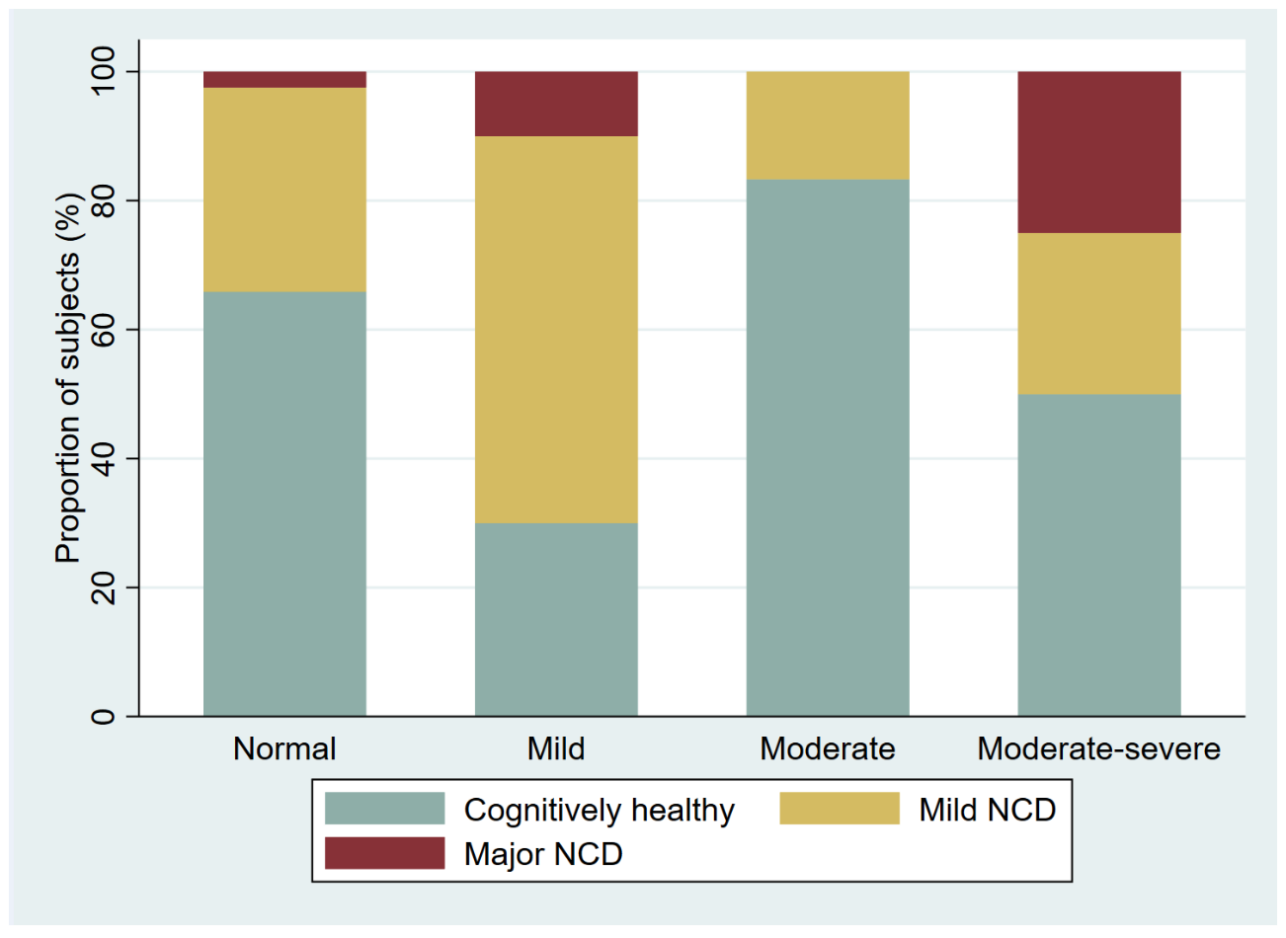
Proportion of cognitively healthy older adults and older adults with possible mild neurocognitive disorder (NCD) and possible Major NCD, per severity of depression symptoms.

**Figure 3:**
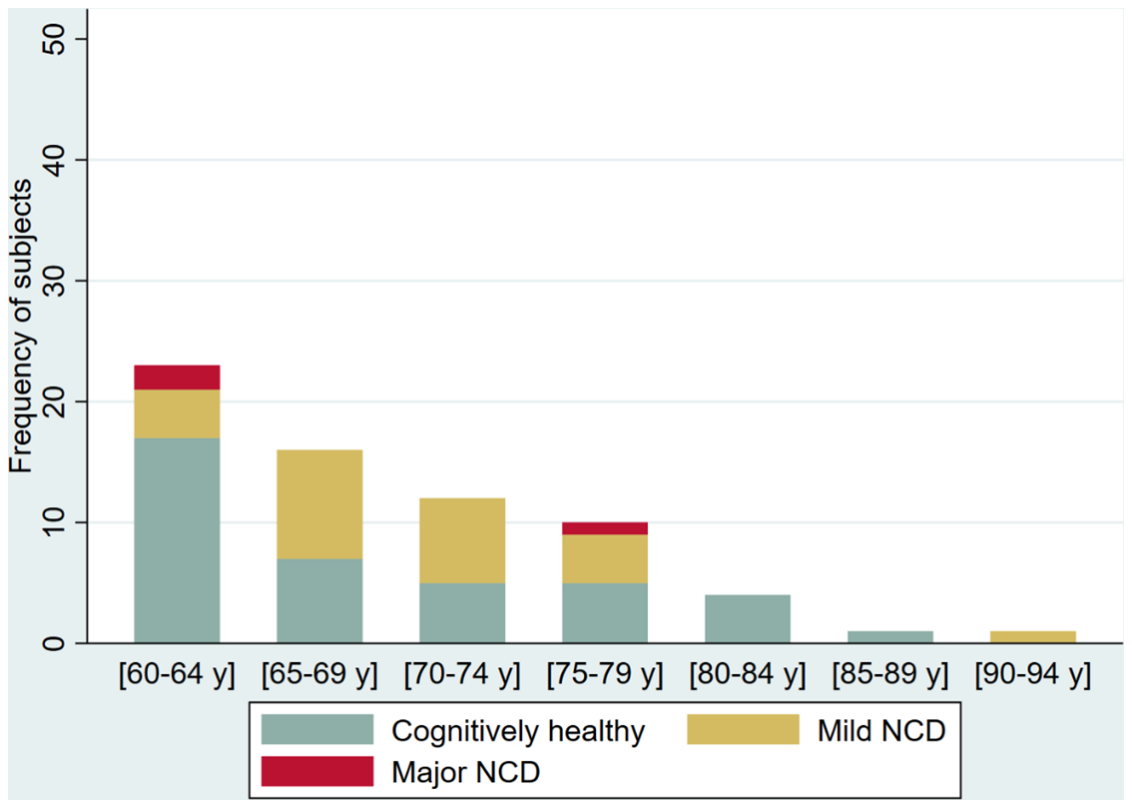
Frequency of cognitively healthy older adults and older adults with possible mild neurocognitive disorder (NCD) and possible Major NCD, per age group.

**Figure 4:**
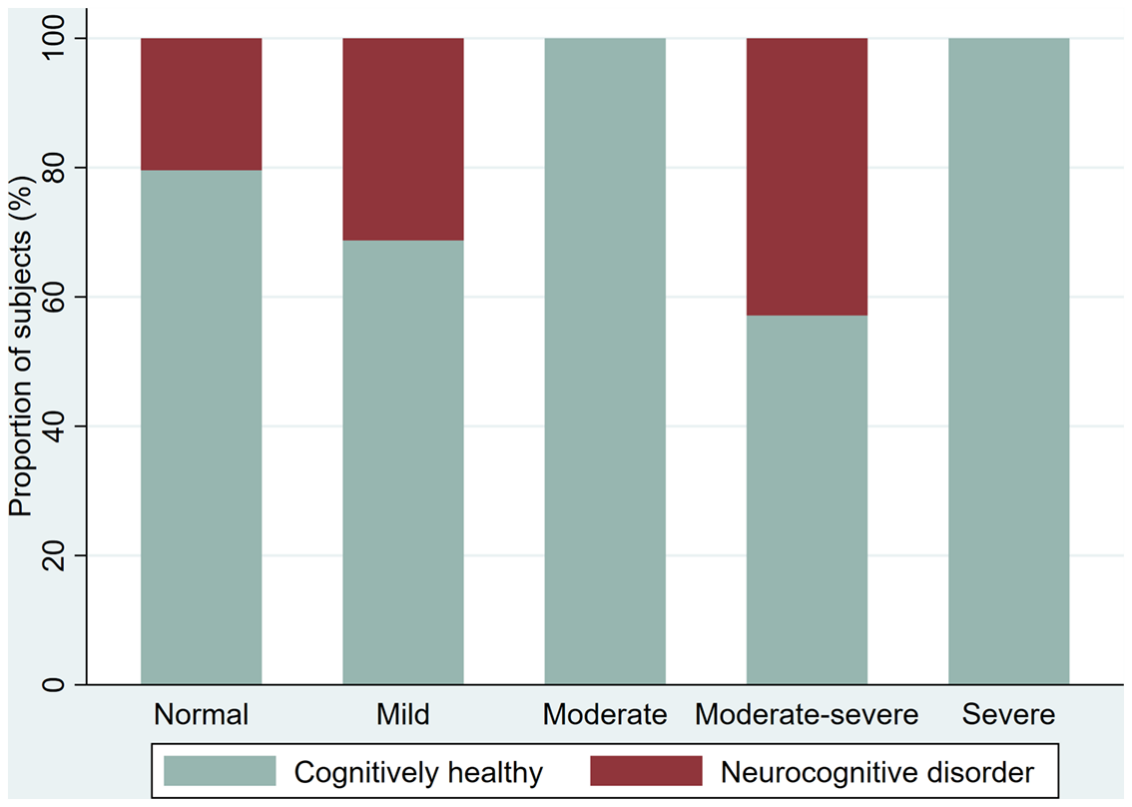
Proportion of cognitively healthy younger adults and younger adults with possible neurocognitive disorder, per severity of depression symptoms.

We screened 221 participants for depression with an overall proportion of 37.1% with depressive symptoms (38.4% in younger adults and 33.9% in older adults). We found the following proportions of depressive symptoms among younger adults: 30.2% with mild depressive symptoms, 3.1% moderate, 3.8% moderate–severe, and 1.3% severe; among older adults, the prevalence of depression symptoms was as follows: 16.1% mild, 9.7% moderate, and 8.1% moderate–severe. We explore the proportion of the NCD by severity of the depressive symptoms (Figures 4 5).

## Discussion

In the COVID-19 pandemic era, this is the first cross-sectional door-to-door pilot study to describe the epidemiology of possible NCDs using validated BCTs in younger and older adult populations from a urban-marginal area in LA. In a sample of 247 participants from an urban-marginalized district of the Peruvian capital city of Lima, we found an NCD prevalence of 25.6% in younger adults and 41.8% in older adults, a finding consistent with other studies conducted in older adults from urban-marginalized communities in Ecuador (30) and Mexico (31), which reported NCD prevalence of 43.9 and 41.7%, respectively.

The results obtained among older adults (≥60 years) differ substantially from those previously reported in door-to-door studies performed in Peru. The first, in 2008, was led by Custodio et al. in an urban community of Lima and found a prevalence of dementia in older adults of 6.85%, with higher prevalence among older age groups, females, and those with low educational levels (32). In 2012, a study by the Dementia Research Group 10/66 reported a crude prevalence of MCI of 3.1% within Peru (33). Finally, in 2012 a study conducted by the National Institute of Mental Health reported a prevalence of MCI and dementia of 6.7% among older adults in Lima and Callao (34). Thus, we believe that our findings suggest a possible increase in the prevalence of NCD in Peru, which is consistent with observed trends in LA (11). Additionally, our findings may differ from NCD prevalence previously reported in Peru conducted in non-marginalized communities given that detrimental socio-environmental factors of urban-marginalized settings may negatively influence brain health and therefore cognitive performance on BCTs. Our study sheds light on the effects that low socioeconomic environments may have on brain health of populations and on the prevalence of NCD in these populations.

However, the difference between our results and previously performed in Peru could be related to the use of diverse methodologies across the different studies, previously highlighted for the LA research in dementia (35), which incurs several limitations. First, these studies utilized instruments that have not been validated for use in Peruvian populations and other specific populations (e.g., the *Mini Mental State Examination* has low sensitivity to detect mild NCD in populations with low educational levels) (24), and with limited criterion validation against gold standard cognitive tests (36). Previous studies also included populations that were not as socioeconomically vulnerable as the population sampled in this study. Moreover, differences in cognitive assessment protocols and other related to evaluator training likely accounted for the low prevalence of NCDs previously reported.

During this pandemic era, Soto et al. carried out a multinational survey in a sample of older adults over 60 years from 10 LA countries, including Peru (37). This study reported a prevalence of dementia lower than our study (23.96%), which could be explained by differences in the studied population (older adults from clinics and centers for older adults), sampling (nonprobabilistic approach), instruments for cognitive screening [telephone version of “Montreal Cognitive Assessment” (T-MoCA) and “Alzheimer Disease 8” (AD8)], and procedures (evaluations were remotely performed using telephone interviews). Moreover, the applied instruments (T-MoCA and AD8) were not validated in the Peruvian population. These methodological differences highlight the need for standardized approaches across community-based prevalence studies for appropriate comparisons between populations.

In the younger adult group (30–59 years), we observed possible NCDs across all five-year age groups, with a significant increase in the oldest five-year age group. This finding is consistent with an increase in the occurrence of dementia in young adults reported in a recent systematic review (11). However, the earlier onset of CI observed could also suggest that even in populations of this age range in which the prevalence of NCDs should be low, the ACE cut-off scores should be further adjusted based on age. During our fieldwork, we detected a consistent difficulty in one of the ACE questions related to recalling the names of government authorities, which may have been due to the high political instability Peru has faced over the last several years (38, 39); this limitation may impact the administration of this test in other equally politically unstable countries in the region.

Previous studies have reported elevated levels of NCDs in younger adults. One such study conducted by Mukaetova-Ladinska et al. found that 30.74% of a sample of 348 young adults from the United Kingdom had suspected dementia (40). Our study findings suggest that urban-marginalized settings may detrimentally affect brain health and mediate lower cognitive performance in younger adults, and perhaps mediate an earlier onset of NCD, as suggested by the peak prevalence observed in the five-year age range of 55–59 years.

Another factor that may explain elevated levels of possible NCDs in our study is COVID-19. Studies have shown that COVID-19 is associated with lower cognitive performance, sustained even after full recovery from the infection (i.e., long COVID) (41). Up to 7.2% of survivors of COVID-19 infection may present with *brain fog*, a generic term used to encompass complaints related to cognitive function among patients with post-acute COVID-19 syndrome, such as vague confusion, impairments across various cognitive domains, and dizziness (42, 43). Post-acute COVID-19 syndrome may have a neurobiological correlate with changes evidenced in quantitative electroencephalograms (44) and brain positron emission tomography scans (45). Several studies have shown that, even in mild cases, COVID-19 can compromise cognitive performance, including inattention, memory impairment, and slower processing speed with improvement months after infection (46–48).

Although older adults were an age-group particularly impacted by the COVID-19 pandemic in LA (49), our study found an unusually low prevalence of NCDs in this group. As the study of Soto et al. (37) and others have shown, our data also suggests that the prevalence of NCDs increases with age, but with a marked decrease among adults age ≥ 80 years. This may be due to lower survival among older adults exposed to the risks of living in marginal urban environments (malnutrition, difficulty in accessing health services, low educational level, poor health literacy, among other health determinants), which results in the high mortality observed in these age groups in LMIC (50, 51) and, particularly in Peru, the country with the highest COVID-19 related deaths per 100,000 population in the world (52). In addition, it is also possible that, compared to healthier participants, mortality risk is greater among those who experience limited independence of ADLs as is seen in older adults with NCDs who have higher mortality risk (53).

Beyond age, other variables such as education, race/ethnicity, and socioeconomic factors may influence the prevalence of NCD in a population (54). Consistent with this, participation in social activities (55) and higher educational levels could be a protective factor against dementia (56, 57), although the high variability of educational quality in LA may lower the protective effects of number of years of education compared with high-income regions (58). In light of the 2020 Lancet Commission report on dementia, which identified 12 modifiable risk factors for dementia that could reduce the incidence of dementia by up to 40% (12), some of our findings may be due to the vulnerability factors unique to the presently studied population and strongly suggest that a careful assessment of social and environmental determinants of health may play an important role in developing NCDs throughout adulthood. These factors should therefore be assessed systematically in observational and epidemiological studies, particularly those social and environmental factors that are most amenable to intervention.

We found that about one-third of participants who completed the PHQ-9 (37.1%) had depressive symptoms classified as mild or worse, with a higher prevalence in young adults (38.4%) than older adults (33.9%). A similar proportion was previously reported in Peruvian older adults from urban areas during the COVID-19 lockdown (38.1%) (59). However, the prevalence of depressive symptoms in our study is lower than that reported in other studies conducted in urban-marginalized settings in LA, such as in Mexico and Ecuador, where a depression prevalence of nearly 60% (31) and 48% (30), respectively, have been reported. The differences between our results and those reported by other studies could be explained by the use of different instruments since these studies applied different versions of the Yesavage geriatric depression scale, a tool with uncertain validity in Peruvian populations (60) and in people from urban-marginalized areas (31).

Mood disorders are common in LMIC, and are difficult to interpret in an epidemiological study such as this one, as relationships between depression and NCDs are complex and difficult to untangle (61, 62). Some studies suggest depression may be a risk factor or early manifestation of neurodegenerative disorders (63, 64), while other studies view depression as risk factor for progression to MCI (61), a predictor of progression from MCI to dementia (65), or a high-prevalence condition in patients with dementia (66). A recent study reported that depressive symptoms not only increase the risk of MCI, but also this MCI could predict subsequent high-risk depression, showing a bidirectional association (67). However, others studies found that depression could predict subjective cognitive impairment, but not objective cognitive impairment (68), and that depression in mid-life is not associated with increased cognitive decline (69). Thus, the current evidence does not support excluding the subjects with probable depression, particularly in a disadvantaged population where at least one per three reported depressive symptoms.

### Implications

Prevalence studies are useful to estimate the magnitude of any health condition, a necessary step towards defining the type and quantity of services needed to address said condition (70). Thus, our findings of a suspected high prevalence of NCDs among younger adults is key to informing decision-making of Peruvian public health authorities and those of neighboring countries in the Andean region. Additionally, our rigorous evaluation approach could be applied for early dementia detection since a recent study showed that an active community-based screening protocol is effective for increasing the rate of early detection up to 3-fold compared to passive screening during routine clinical practice (71). Moreover, we would need to further look at the association between depression and NCD in our study population to determine the influence of depression on NCD prevalence. To-date, there are no reported initiatives for community-based dementia screening protocols in LA.

### Limitations and strengths

Due to the complexities inherent in conducting a community-based study in vulnerable settings and limitations of resources for our study, it was not possible to perform a complete medical evaluation to determine the specific etiology of the observed NCDs (i.e., vascular dementia, suspected Alzheimer’s disease, etc.) or to apply complete neuropsychological assessments in the home of participants. Moreover, we were unable to perform a functional assessment on most participants in this study due to the absence of an informant in the home or, when an informant was available, their refusal to participate in the study. Hence, possible NCD cases were defined solely on the basis of BCT results, as has been done in other population-based studies (30, 31, 72) and clinical investigations (73) and is consistent with diagnostic approaches that do not require an assessment of the patient’s functionality (74). In future studies, we will work to find ways of incorporating brief functional evaluations into our study protocol in a more effective manner.

With respect to the younger adult group (30 to 59 years of age), given the lack of tests specifically designed to assess NCDs in this age range, we used the ACE because there is some evidence to support its use in younger ages based on prior studies (75, 76). Moreover, not all participants completed the PHQ-9, and we did not exclude participants who had depressive symptomatology given a high co-existence of dementia and depression, as has been previously observed in another study that demonstrated 36.7% of patients with dementia had depressive symptomatology (77). Among patients with dementia living in a residential home, nearly 60% had moderate or worse depressive symptoms (78). Therefore, given depression and NCD highly correlate with one another, it is crucial to consider these patients in dementia studies.

The present study has several strengths. First, we conducted cognitive assessment among older adults with an instrument (RUDAS-PE) that has been widely validated in Peru for various educational levels, including low educational levels and illiteracy (23–25). In contrast to other door-to-door studies in LA that have used non-adapted and non-validated BCTs (79), we used a validated tool. Our study is the first to include younger adults (30 to 59 years), notably below previously considered age ranges (56). Lastly, the field team of evaluators underwent a rigorous training process to ensure standardized and harmonized administrations of all field testing, not a small feat considering that this study occurred during the COVID-19 pandemic in one of the countries most highly affected by the pandemic.

## Conclusion

In our study of 247 subjects from the urban-marginalized district of Puente Piedra, we found a prevalence of possible NCDs of 30%, with 25.6 and 41.8% prevalence among younger adults and older adults, respectively. Among older adults, we identified a low prevalence of possible NCDs, while among younger adults we identified high levels of possible NCD, with a peak in the 55–59 years age group, which could suggest a relatively early onset of possible NCDs in populations as socioeconomically vulnerable as our study population.

This study supports the feasibility of the broad use of brief cognitive tests, such as the RUDAS-PE, not only for field research purposes, but also for public health research or health policy evaluations. We recommend that community-based prevalence studies (e.g., demographic and health surveys) use BCTs validated in the targeted study populations. Similarly, such tests should be incorporated into the arsenal of screening tools at the primary health care level for the implementation NCD detection protocols and the development of referral processes to specialists at the primary care level.

Our findings in younger adult populations should be corroborated with future larger studies that consider more rigorous validation processes for the detection of NCDs in these age groups. If corroborated, health systems could incorporate early screening programs for NCD in young age, and the most appropriate *timing* for interventions should be determined. Clinical practice guidelines and healthcare public policies should include recommendations on management of NCDs among younger adults, including validation of BCTs for NCD screening, as well as the diagnosis and management of NCDs among younger populations.

## Data availability statement

The original contributions presented in the study are included in the article/supplementary material, further inquiries can be directed to the corresponding author.

## Ethics statement

The studies involving humans were approved by ethics committee from the Universidad San Ignacio de Loyola. The studies were conducted in accordance with the local legislation and institutional requirements. The participants provided their written informed consent to participate in this study.

## Author contributions

EH-P, NC, RM, MD, MV, and SL designed the study. EH-P, and MV supervised the data collection. AC collected the data. EH-P, MD, and AC were responsible for the statistical design of the study and for carrying out the statistical analysis. EH-P, NC, MD, and SL wrote the manuscript. All authors critically reviewed the manuscript and approved the submitted version.
